# ADVANCING POSITIVE SOCIAL CONNECTIONS FOR OLDER ADULTS IN SUBSIDIZED HOUSING: A HUMAN-CENTERED DESIGN APPROACH

**DOI:** 10.1093/geroni/igad104.3754

**Published:** 2023-12-21

**Authors:** Sheena Pubien, Becky Slogeris, Elizabeth Bazurto, Kennedy McDaniel, Vilde Ulset, Carl Latkin, Cynthia Boyd, Thomas Cudjoe

**Affiliations:** Nova Southeastern University, North Lauderdale, Florida, United States; Maryland Institute College of Art, Baltimore, Maryland, United States; Maryland Institute College of Art, Baltimore, Maryland, United States; Johns Hopkins University, Baltimore, Maryland, United States; Maryland Institute College of Art, Baltimore, Maryland, United States; Johns Hopkins Bloomberg School of Public Health, Baltimore, Maryland, United States; Johns Hopkins School of Medicine, Baltimore, Maryland, United States; Johns Hopkins University School of Medicine, Baltimore, Maryland, United States

## Abstract

Social isolation is a complex problem that impacts many community-dwelling older adults and has detrimental impacts on mental and physical wellbeing of older adults. This research aims to utilize Human-Centered Design (HCD) to develop and implement tools and design ideas that advance positive social connections of older adults residing in subsidized housing. HCD is a collaborative process used to understand and define social problems, identify opportunities, generate ideas, and design interventions to support positive change. We sought to capture the perspectives and insights from older adults living in two federally subsidized housing communities in Baltimore, Maryland. During a five-month period, we held listening and brainstorming sessions with over 50 residents, service coordinators and members of management. Valuable insights and ideas were recorded during each of these facilitated engagements and synthesized. Through our engagements we identified six themes that influence the social connections of older adults living in subsidized housing: feelings of exclusion, lack of community connections, burdensome transitions to communal living, changes to building policies, and concerns about the physical environment. These themes led to the creation of interventions such as providing physical materials to residents, facilitating cultural inclusion and connections, implementing effective engagement strategies for community coordinators and promoting resident leadership. HCD can be employed to develop tools and innovative opportunities that specifically target and address social isolation in older adults living in subsidized housing. HCD can be utilized to drive changes to policies that impact older adults in subsidized housing leading to social transformations to improve health.

